# Computer modeling and ex vivo experiments with a (saline-linked) irrigated electrode for RF-assisted heating

**DOI:** 10.1186/1475-925X-13-164

**Published:** 2014-12-12

**Authors:** Javier Arenas, Juan J Perez, Macarena Trujillo, Enrique Berjano

**Affiliations:** Biomedical Synergy, Electronic Engineering Department (Building 7 F), Universitat Politècnica de València, Camino de Vera 46022, Valencia, Spain; Bioelectronic Research Group (I3BH) (Building 7 F), Universitat Politècnica de València, Camino de Vera 46022, Valencia, Spain; Instituto Universitario de Matemática Pura y Aplicada, Universitat Politècnica de València, Camino de Vera 46022, Valencia, Spain

**Keywords:** Computer modeling, Irrigated electrode, Mathematical modeling, Radiofrequency ablation, Radiofrequency-assisted resection, Saline-linked technology

## Abstract

**Background:**

Externally irrigated radiofrequency (RF) electrodes have been widely used to thermally ablate tumors in surface tissue and to thermally coagulate the transection plane during a surgical resection. As far as we know, no mathematical model has yet been developed to study the electrical and thermal performance of these electrodes, especially the role of the saline layer that forms around the electrode.

**Methods:**

Numerical models of a TissueLink device model DS3.0 (Salient Surgical Technologies, Portsmouth, NH, USA) were developed. Irrigation was modeled including a saline layer and a heat convection term in the governing equation. Ex vivo experiments based on fragments of bovine hepatic tissue were conducted to obtain information which was used in building the numerical model. We compared the 60°C isotherm of the computer results with the whitening contour in the heated samples.

**Results:**

Computer and experimental results were in fine agreement in terms of lesion depth (2.4 mm in the simulations and 2.4 ± 0.6 mm in the experiments). In contrast, the lesion width was greater in the simulation (9.6 mm vs. 7.8 ± 1.8 mm). The computer simulations allowed us to explain the role of the saline layer in creating the thermal lesion. Impedance gradually decreased as heating proceeded. The saline was not observed to boil. In the proximity of the electrode (around 1 mm) the thermal lesion was mainly created by the RF power in this zone, while at a further distance the thermal lesion was created by the hot saline on the tissue surface by simple thermal conduction. Including the heat convection term associated with the saline velocity in the governing equation was crucial to verifying that the saline layer had not reached boiling temperature.

**Conclusions:**

The model reproduced thermal performance during heating in terms of lesion depth, and provided an explanation for: 1) the relationship between impedance, electrode insertion depth, and saline layer, and 2) the process of creating thermal lesions in the tissue with this type of electrode.

## Background

Medical devices based on radiofrequency (RF) energy are usually employed during surgical resection to thermally coagulate tissue in order to minimize intraoperative blood loss. The rationale of applying RF power is to achieve sufficiently deep thermal lesions so as to seal the small vessels in the transection plane. Some of these devices are based on irrigated electrodes, which infuse saline into the tissue through openings [1]. Although electrodes with this type of irrigation have been broadly used in other clinical procedures, such as RF cardiac [2] and tumor [3] ablation, in the context of RF-assisted surgical resection they are referred to as *saline-linked* RF electrodes [1]. There are mainly two clinically available arrangements: monoplar and bipolar. The monopolar is commercially available in the form of the TissueLink device (Salient Surgical Technologies, Portsmouth, NH, USA) which is a dissecting sealer initially proposed to ablate surface tumors [4], and later employed in numerous RF-assisted surgical resection procedures in different organs such as spleen [5, 6] and pancreas [7, 8]. The bipolar arrangement is a recent proposal and is available in the form of the Aquamantys device (Medtronic, Minneapolis, MN, USA), which is intended to reduce blood loss during hip arthroplasty [9], hepatic resection [10] and spinal surgery [11]. In both arrangements the saline irrigation is aimed at preventing surface charring and keeping the tissue surface temperature below 100°C [4].

Despite their extensive clinical use, to our knowledge, no numerical models have been proposed to describe the performance of saline-linked RF electrodes. From an engineering point of view it is important to achieve a physical description in the form of the mathematical relations between the electrical and thermal variables involved. This would provide further information on, for instance, the role of the saline layer in creating thermal coagulation. Our aim in the present work was to develop a numerical model to describe the electrical and thermal performance of a monopolar saline-linked RF electrode designed to heat the tissue surface. In clinical terms this means to thermally coagulate the transection plane during a surgical resection or to thermally ablate surface tumors.

## Methods

The building of the numerical model was supported by experiments conducted on an ex vivo model. Specifically, the experimental results were used to 1) adjust some of the characteristics of the numerical model, and 2) validate the computer results.

### Geometry of the numerical model

**Figure 1:**
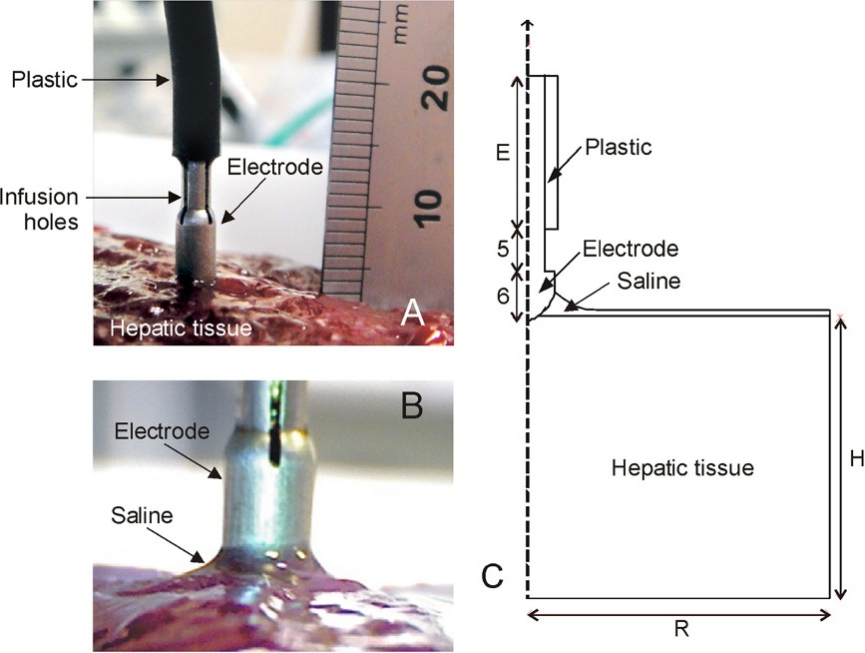
**Physical situation and theoretical model. A**: Physical situation with the electrode placed perpendicular to the tissue surface before saline infusion. **B**: Detail of the saline layer around the electrode. **C**: Geometry of the theoretical model (out of scale, dimensions in mm).

Although saline-linked RF electrodes can be used in almost every position relative to the tissue surface to be treated, we simplified the physical situation by considering an electrode placed perpendicularly to the tissue surface (Figure 1). The problem consequently presented axial symmetry and a two-dimensional analysis was possible, which reduces the computational requirements. The computer results were compared to those obtained from a bench-test based on an ex vivo hepatic tissue model. We took into account some of the characteristics of the experimental set-up during the creation of the mathematical model. The idea behind this was to keep the effect of certain variables under control as far as possible. Figure 1A and B show the physical situation of the experiments, and Figure 1C shows the geometry of the proposed model. In the numerical model the tissue was considered to be a cylindrical volume with the same dimensions as the volume of hepatic tissue used in the experiments: 65 mm radius (R) and 60 mm high (H). The electrode length E was 10 mm. Likewise, since the dispersive electrode in the experiments was a circular metallic plate of 65 mm radius placed under the tissue fragment, it was modeled as an electrical condition on the bottom boundary. We considered a Model DS3.0 TissueLink device (Salient Surgical Technologies, Portsmouth, NH, USA) of 3 mm in diameter, whose tip was modeled with its real dimensions (Figure 1C). As the three holes placed symmetrically on the lateral surface through which the saline is infused onto the tissue surface were not considered in the model, it was hence assumed that the saline is infused through the entire lateral surface of the tip. The realistic modeling of the saline layer over the tissue is addressed below.

### Governing equations of the numerical model

The numerical model is based on a coupled electric-thermal problem. The governing equation for the thermal problem was the bioheat equation modified by the enthalpy method to take into account the vaporization phenomenon [12]:
1

where *ρ* is density, *h* enthalpy, *T* temperature, *t* time, *k* thermal conductivity, and *Q*_*p*_ and *Q*_*m*_ are the terms corresponding with blood perfusion and metabolic heat respectively. Since the computer results were compared with experimental results obtained from an ex vivo model, *Q*_*p*_ and *Q*_*m*_ were set to be zero. Although in order to model a real scenario, additional computer simulations were conducted including the blood perfusion term as follows:
2

where *ρ*_*b*_ is density of blood (1000 kg/m^3^), *c*_*b*_ specific heat of blood (4180 J/kg · K), *T*_*b*_ blood temperature (37°C), *ω*_*b*_ blood perfusion coefficient (6.4 × 10^-3^ s^-1^) [13] and *β* is a coefficient which took the values of 0 and 1, depending on the value of the local thermal temperature: *β* = 0 for temperature ≥ 50°C, and *β* = 1 for temperature < 50°C. In these additional computer simulations the initial temperature was assumed to be 37°C.

The heat source *q* was obtained from the electrical problem with *q* = *σ* ⋅ *E*^2^, where *σ* is the electrical conductivity and *E* is an electric field, which is obtained from *E* = - ∇*V* where *V* is the voltage. This voltage was obtained from the Laplace equation ∇ ⋅ *σ*∇*V* = 0, which was the governing equation of the electrical problem. At RF frequencies (≈500 kHz) and over the distance of interest, the biological medium can be considered almost totally resistive and a quasi-static approach is therefore possible to solve the electrical problem [14].

### Characteristics of the materials of the numerical model

Table 1 shows the characteristics of the materials considered in the theoretical model [15–17]. The dependence of the electrical conductivity of tissue and saline with temperature was modeled using a piecewise function [13]:

**Table 1:** **Characteristics of the materials considered in the theoretical model [** [15]**-** [17]**]**

Material	Thermal conductivity	Specific heat	Density	Electrical conductivity
	***k***(W/m⋅K)	***c***(J/kg⋅K)	***ρ***(kg/m ^3^)	***σ***(S/m)
Plastic	0.026	1045	70	10^-5^
Electrode	70	840	6450	10^8^
Saline	0.58	3200	1004	*a* ^(3)^
Hepatic tissue	0.502	3455 ^(1)^	1080 ^(1)^	*b* ^(3)^
		2155 ^(2)^	370.44 ^(2)^	

^(1)^Tissue in liquid phase. ^(2)^Tissue in gas phase. ^(3)^The values of electrical conductivity of tissue and saline were estimated from experimental results (see text for further details).

3

where *i* = saline, tissue. The values of *σ*_*i*_(37°C) for tissue and saline were estimated from experimental results in order to reduce the variability associated with this parameter. For hepatic tissue the first term in equation (3) was computed as:
4

where the subscript *l* refers to the liquid tissue phase and *g* to the gas tissue phase (see Table 1), *c* is the specific heat, *H* is the product of the water latent heat (*H*_*lg*_ = 2.582 kJ/kg · K) and the percentage of water content in hepatic tissue (68%) and *ρ*_*m*_ is the water density at 99°C (958 kg/m^3^).

In the case of the saline layer, we assumed that vaporization is permanently compensated by the continuous supply of liquid and hence the gas phase is never reached. We mathematically modeled this phenomenon by considering a latent heat which is kept constant after 99°C:
5

where *ρ*_*saline*_ and *c*_*saline*_ are saline density and specific heat respectively (see Table 1). In order to check whether this approach significantly affected the results, an additional simulation was conducted assuming that the saline thermal performance was identical to that of tissue beyond 99°C.

### Boundary and initial conditions for electrical and thermal problem

The electrical boundary conditions were of electrical insulation on the entire plastic and tissue surfaces, excluding the lower tissue surface, on which the condition was *V* = 0 V (dispersive electrode). A constant voltage of 47 V was applied to all electrode surfaces, a value identical to that used in the experiments. This value corresponds with the root-mean-square value of the RF voltage applied during the experiments.

The thermal boundary conditions were of thermal isolation on the lateral tissue surfaces (since in the experiments the tissue fragment was inserted into a plastic cylindrical container), and natural convection on all the outer electrode and plastic surfaces and the upper tissue surface. Natural convection was modeled by a heat flow (*q*_*c*_) following Newton’s cooling Law:
6

where *h*_*e*_ is the free convection thermal coefficient which was assumed to be 20 W/m^2^⋅K, and *T*_*a*_ is the ambient temperature (measured during experiments). The initial electrode and tissue temperature was equal to that measured in the experiments.

### Modeling of saline layer

**Figure 2:**
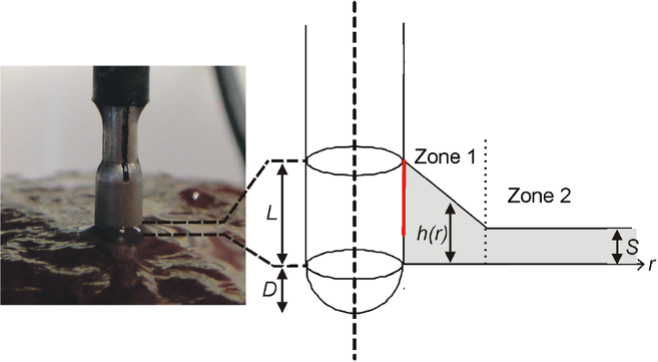
**Modeling of the geometry of saline layer around electrode.** Estimation of the geometry of the saline layer (grey zone) around the electrode surface (out of scale). The height (h) of the layer depends on the distance to the electrode surface (r). The electrode is assumed to be inserted into the tissue to depth D. The saline velocity distribution in the layer was calculated by solving the Navier–Stokes equations. A boundary condition of constant velocity was set on a specific zone of the electrode surface (red line).

Images of the saline distribution around the electrode were taken with a USB-microscope (200×) and dimensionally analyzed (0.5 mm resolution ruler) to build the estimated geometry of the saline layer around the electrode. We observed that this layer was created with a more or less exponential decay next to the electrode and a more or less constant thickness away from it (Figure 1B). Accordingly, we simplified the geometry of the saline layer by considering two zones as shown in Figure 2. Zone 1 has a linear drop (which is an approximation of the exponential decay) and Zone 2 has a constant thickness *S* = 0.6 mm. The height of the saline layer on the electrode surface was *L* = 1.5 mm and the *extension* of the zone 1 was 2 mm (*r*_*0*_).

The thermal effect caused by the flow of saline was considered by introducing a new term in equation (1):
7

where u is saline velocity (m/s). The distribution of saline velocity u was calculated using Navier–Stokes equation [18]. A boundary condition of constant velocity was set at 5.3 · 10^-3^ m/s on a specific zone of the electrode surface (red line in Figure 2). This value was obtained as the ratio between the flow rate *Q* and the electrode area through which saline flows (9.4 · 10^-6^ m^2^). *Q* was identical to that clinically employed, i.e. one droplet per second. By considering 20 droplets equivalent to 1 mL, *Q* = 3 mL/min (5 · 10^-8^ m^3^/s). We assumed a wall condition (no slip) at the saline-tissue interface and open boundary condition at the saline surface.

Additionally, in order to check the thermal effect of the saline flow (term **u** in the equation (7)), we conducted a computer simulation in which the saline velocity was reduced to zero.

### Meshing and numerical model solver

The model mesh was heterogeneous, with a finer mesh size at the electrode-tissue interface, where the highest electrical and thermal gradients were expected. The size of the finer mesh was estimated by a convergence test. We used the value of the maximum temperature (T_max_) reached in the tissue after 10 s of RF heating as a control parameter in these analyses. When there was a difference of less than 0.2% in T_max_ between simulations we considered the former mesh size as appropriate. Analogously, we used a convergence test to estimate the optimal time-step and the length E of the model.

The model was solved numerically by the Finite Element Method using COMSOL Multiphysics UMFPACK Solver software (COMSOL, Burlington MA, USA). Simulations were run on a PC 64-bit dual-core Intel Xeon at 2.67 GHz and with 48 GB RAM running on a Windows 7 Professional 64 bit operating system.

### Experimental setup

The experimental model was based on cylindrical fragments of bovine liver (130 mm diameter and 65 mm thick) inserted into a plastic container, with a metallic circular piece underneath acting as dispersive electrode. Since preliminary experiments showed that the pressure between electrode and tissue was a critical parameter, we set up a tool to keep the pressure conditions constant. The Tissuelink device is usually connected to a conventional electrosurgical unit in coagulation mode, whose output is a high-voltage signal with a waveform based on bursts of damped sinusoidal signal. However, in this study, in order to match the experimental and computer modeling conditions, we used an RF generator model CC-1 (Radionics, Burlington, MA, USA) which provides a non-modulated sinusoidal signal. In spite of this, we consider that the characteristics of the electrical excitation source should not invalidate the usefulness of the proposed model or its conclusions.

The electrical impedance evolution was provided by the RF generator itself as an analog signal which was sampled (10 Hz) and digitalized with a USB-1208LS data acquisition card (Measurement Computing, Norton, MA, USA). The flow rate of 3 mL/min in the Tissuelink device was set by a syringe pump KDS-200-CE (KdScientific, Holliston, MA, USA). The experiments were conducted at room temperature (24°C) and tissue fragments were likewise at 24°C.

#### Experiments for the adjustment of the electrical characteristics and validation of geometry

The *σ*_*i*_(37°C) of saline and tissue considered in the modeling (see equation (2)) was estimated from experiments. Impedance measurements were first taken with the electrode without saline infusion and completely inserted into a fragment of tissue at room temperature. The obtained values were compared to those obtained from computer simulations where the same setup was modeled with COMSOL Multiphysics, which allowed us to estimate the value of *σ*_*saline*_(37°C). Impedance measurements were also taken from the electrode without saline infusion and completely immersed in a saline tank at room temperature in order to estimate the electrical conductivity of the saline *σ*_*tissue*_(37°C).

Once the values of *σ*_*i*_(37°C) for tissue and saline had been separately obtained, additional experiments were conducted to measure the impedance values of the electrode placed on the tissue both with and without a saline layer. The obtained values were compared to those from the computer simulations, which allowed us to validate indirectly the geometry of the model, in particular the estimation of the insertion depth *D* and the dimensional characteristics of the saline layer (Figure 2).

#### Experimental setup for RF heating

Ten thermal lesions were created by applying 47 V for 20 s. After heating, each lesion was cross-sectioned and photographed to measure the depth and width of the white zone using a ruler (0.5 mm resolution). The whitening contour was assumed to correspond approximately with the 60°C isotherm [19]. This isotherm was exclusively used to compare the experimental and computational results, but not to estimate the irreversibly damaged tissue zone, since it is known that the critical temperature for this state after a hyperthermic exposure of several seconds is around 50°C [20].

## Results

### Experimental results

The first set of experiments was intended to measure the electrical conductivities of tissue and saline. The impedance measured experimentally when the electrode was completely inserted into the tissue (without saline infusion) was 164 ± 6 Ω (n = 3), which allowed us to estimate a *σ*_*tissue*_(37°C) value of 0.31 S/m. Likewise, the experimental value of the impedance in the case of the electrode completely submerged in saline was 65 Ω (at 20°C), from which we estimated a *σ*_*saline*_(37°C) value of 0.774 S/m.

**Figure 3:**
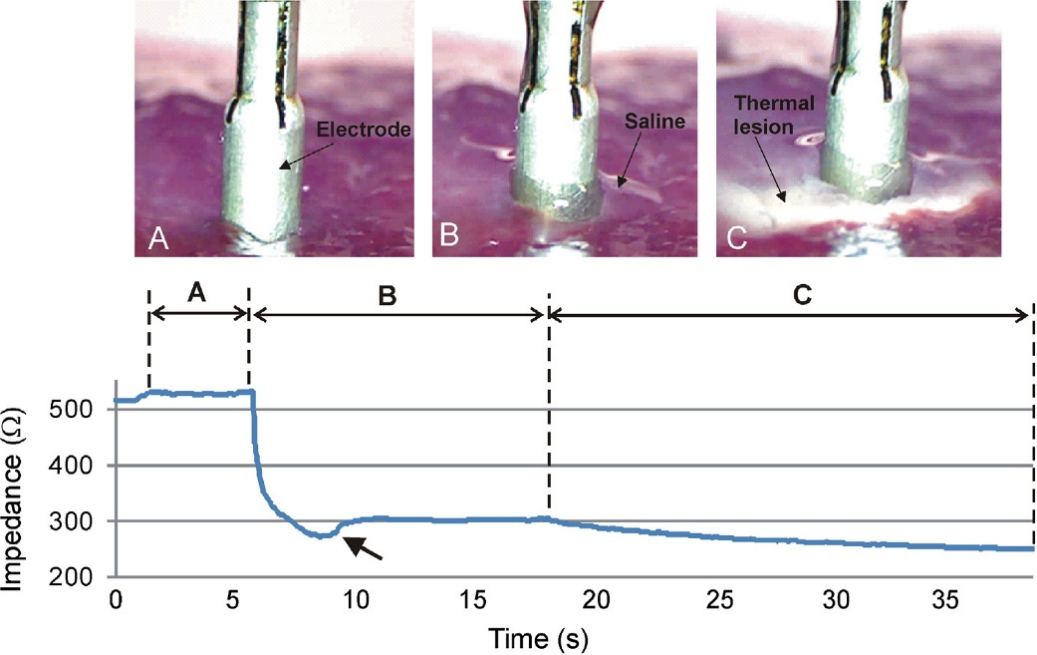
**Impedance progress throughout the experiments.** Three phases can be observed: **A)** Electrode placed on tissue surface without saline infusion. **B)** Saline is infused and impedance drops drastically; the saline initially accumulates around the electrode, forming a pool (i.e. the height h of the saline layer increases considerably) possibly due to the depression caused by the pressure of the electrode on the tissue. Some seconds after continuous infusion sets the retained saline in motion the pool disappears, the height h decreases slightly and consequently impedance rises (arrow) until reaching a steady state. **C)** During RF heating tissue temperature increases gradually and impedance decreases in proportion.

A second set of experiments was conducted with two objectives. The first was to measure the impedance values of the electrode placed on the tissue with and without a saline layer, and in this way corroborate the assumptions on the insertion depth of the electrode and geometry of the saline layer. The second objective was to assess the capability of the numerical model to predict both the impedance progress during RF heating and the thermal lesion size. For these purposes, we conducted consecutive impedance measurements under different conditions (see Figure 3): electrode placed on the tissue without saline layer (Phase 1), electrode placed on the tissue with saline layer (Phase 2), and RF heating (Phase 3).

In Phase 1 (Figure 3A), the electrode was placed on the tissue surface without saline infusion. The impedance measured under these conditions was 563 ± 25 Ω (range 525–597 Ω). In Phase 2 (Figure 3B), impedance dropped drastically. In some cases, the saline initially accumulated around the electrode in a pool, (i.e. the height *h* of the saline layer increased considerably, see Figure 2). This was possibly facilitated by the depression caused by the pressure of the electrode on the tissue. Some seconds later the continuous infusion set the retained saline in motion, the pool disappeared, the height *h* decreased slightly, and consequently impedance increased (see arrow in Figure 3) until reaching a relatively steady state with a value of 310 ± 15 Ω (range 277–330 Ω).

**Figure 4:**
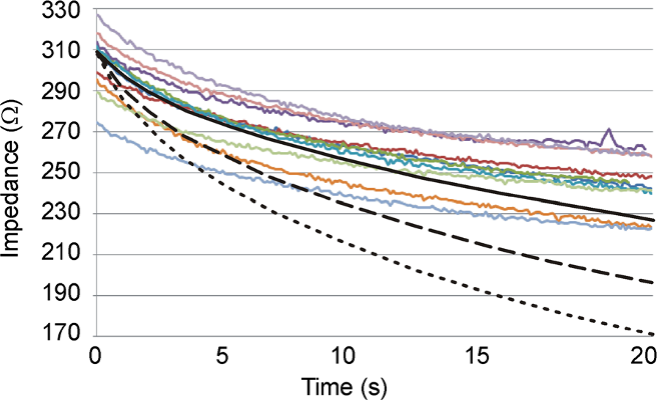
**Impedance progress during RF heating.** Colored solid lines correspond with the experimental results. Black lines correspond with the computer simulations for different values of temperature dependence of electrical conductivity: +1%/°C (solid), +1.5%/°C (dashed) and +2%/°C (dotted). Note that a value of +1%/°C allowed to match computer and experimental results.

**Figure 5:**
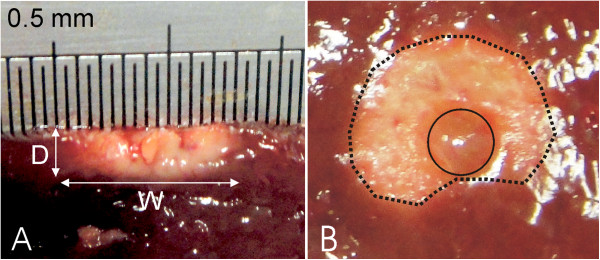
**Thermal lesions created after RF heating.** Side **(** **A)** and surface **(B)** view of the thermal lesions created. After RF heating each lesion was cross-sectioned and the side view was photographed. On each side view, depth (D) and width (W) was measured. As observed in the surface view, the thermal lesions were generally non symmetrical with respect to electrode location (solid line), showing a non circular surface lesion (dotted line).

In Phase 3 (Figure 3C), RF power was applied and consequently a thermal lesion was created in the tissue beneath the electrode. During this phase impedance gradually decreased due to the rise in tissue temperature. Figure 4 shows the impedance progress during the 20-second RF heating phase. In all cases, regardless of the specific impedance value at the beginning of the heating phase, the progress was similar, with a drop of 60 ± 7 Ω (range 50–73 Ω), i.e. the impedance at the end of the RF heating was 244 ± 13 Ω (range 223–263 Ω). The saline was not observed to boil in any case during heating. No audible popping occurred (associated with overheating in deep zones of the tissue). Figure 5 shows a view of the thermal lesions created. The width of the thermal lesions (assessed by the whitening contour) was W = 7.8 ± 1.8 mm, and the depth was D = 2.4 ± 0.6 mm (Figure 5A). Most of the lesions were not perfectly circular on the surface (see Figure 5B).

### Numerical results

The convergence tests provided an electrode length of E = 10 mm and a time step of 0.1 s. The numerical model had 7,280 elements. Although the tissue dimensions R and H were chosen to be identical to the dimensions used in the experiments (R = H = 65 mm), the convergence test provided a value of R = H = 50 mm, which implies that the electrical boundary conditions (associated with the plastic sheath and dispersive electrode) were applied sufficiently far from the active electrode. Once the *σ*_*tissue*_(37°C) was estimated to be 0.31 S/m, we computed the impedance of the electrode placed on the tissue with no saline layer, in order to estimate the insertion depth of *D*. We obtained a value of 0.75 mm, which provides an impedance of 561 Ω, very close to the experimental value of 563 ± 25 Ω.

Computer simulations of the RF heating were conducted for three values of the temperature dependence of the electrical conductivity (tissue and saline): +1%/°C, +1.5%/°C and +2%/°C. Figure 4 shows the impedance progress of the computer simulations (black lines). Although a value of +1.5%/°C had typically been considered in previous studies, we found that it was only possible to obtain a theoretical result similar to the experiments with a value of +1%/°C, as all others caused the impedance progress to drop too fast.

**Figure 6:**
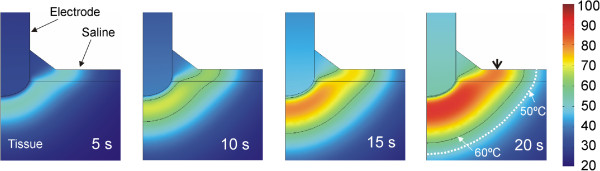
**Evolution of the temperature distributions during RF heating (scale in °C).** Black line represents the 60°C isotherm which is used for comparison with the whitening contour observed in the heated samples in the experimental model. Note that 1) no boiling temperatures (around 100°C) are observed in the saline layer, and 2) the heating in the tissue far from the electrode is mainly caused by the heated saline flowing over it (arrowhead). In other words, the tissue temperature is higher than the saline temperature beneath the electrode, whereas the saline temperature is higher than the tissue temperature at distant points (white line represents the 50°C isotherm).

Figure 6 shows the temperature distributions throughout the 20 seconds of RF heating using the value of +1%/°C. No boiling temperatures (around 100°C) were observed in the saline layer. The thermal lesion was mainly created underneath the electrode due to the RF power being mostly deposited in that zone. Although RF currents flowed through the saline, the saline temperature was always lower than that in the tissue, at least next to the electrode. On the other hand, as shown in Figure 6, the saline temperature was higher than the tissue temperature at points distant from the electrode, which suggests that the heating in the tissue away from the electrode could be mainly caused by the pre-heated saline flowing over it. The 60°C isotherm in the tissue computed from simulations was 2.4 mm in depth and 9.6 mm in width (surface). While the depth value is in close agreement with that obtained from the experiments (2.4 ± 0.6 mm), the computed width value is higher than the experimental value (7.8 ± 1.8 mm).

**Figure 7:**
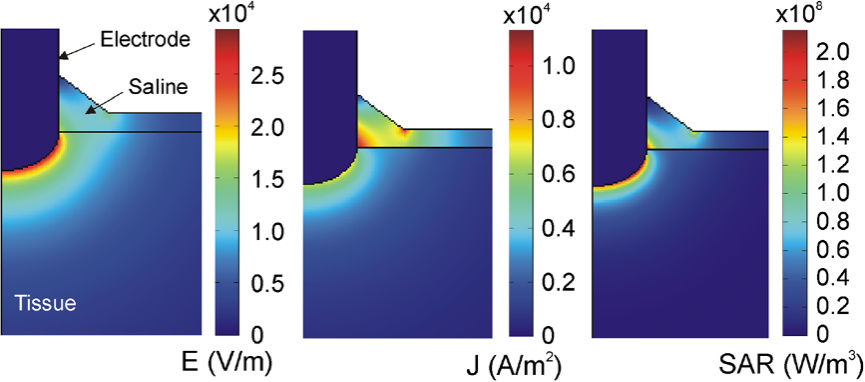
**Spatial distribution of the electrical variables.** Electric field (E), current density (J) and Specific Absorption Rate (SAR) at the beginning of the RF heating (after 1 second).

In order to gain a better understanding of the temperature distributions shown in Figure 6, we analyzed in detail the electrical variables involved in the RF power application. Figure 7 shows the spatial distribution of the electric field (*E*), current density (*J*) and Specific Absorption Rate (SAR) at the start of heating (at 1 second). The electric field distribution in the tissue was more or less predictable, showing a high gradient next to the electrode’s spherical zone. The current density showed higher values in the saline region, due to the fact that the value of *σ* is higher in this zone than in the tissue. As a consequence, the applied power density (SAR) was mainly located in the tissue next to the spherical section of the electrode, and to a lesser extent at some points in the saline, especially where the current density was maximal. This actually matches with the temperature distributions shown in Figure 6, where the main temperature increase is located in the tissue under the electrode and is lower in the saline.

**Figure 8:**
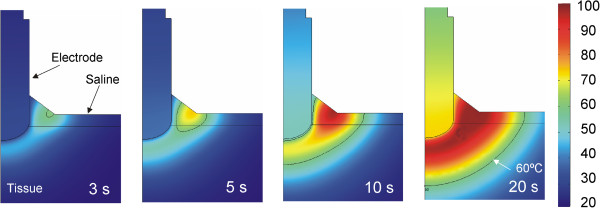
**Evolution of the temperature distributions without saline motion.** Evolution of the temperature distributions (scale in °C) during RF heating considering that saline velocity is zero. Note that in this case boiling temperatures (around 100°C) appear in the saline.

In order to assess the thermal effect of the saline flow (term u in the equation (7)), we conducted an additional computer simulation in which the saline velocity was reset to mimic a drastic reduction of the infusion in practical terms. Figure 8 shows the evolution of the temperature distributions during RF heating in this case. The thermal performance is clearly very different to that in which the saline is in motion. The differences are: 1) the temperature rise is initially located in the saline (and not in the tissue), and 2) the maximum temperature is mainly located in the saline throughout the heating, even reaching 100°C in the saline surface, The electrical performance was identical in both cases and corresponds with that shown in Figure 7.

One of the original issues in this work was the mathematical approach used to model the thermal effect of the continuous supply of saline around the electrode (equation (5)). In order to check whether this approach significantly affected the results, an additional simulation was conducted assuming that the saline thermal performance was identical to that of the tissue beyond 99°C (i.e. in accordance with equation (4)). The results were identical in both cases, which can be explained by the saline temperature not reaching this value (was always lower than 85°C), so that the modified performance of the saline in the new mathematical approach did not work in this case. In other words, the movement of the saline made the tissue temperature stay below 100°C, so that the mathematical approach did not influence this case.

**Figure 9:**
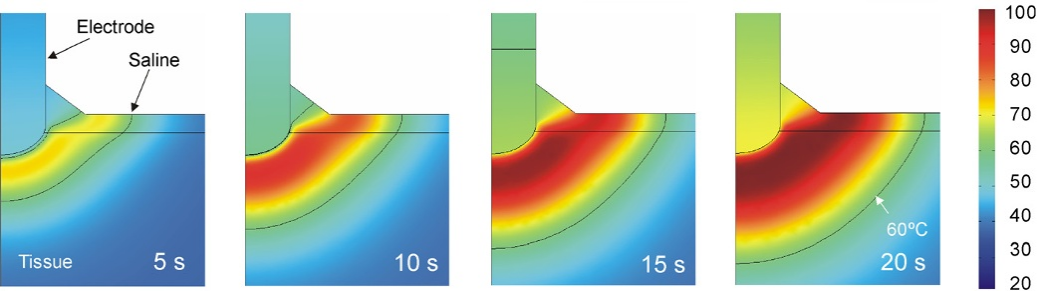
**Evolution of the temperature distributions in the case of in vivo situation.** Evolution of the temperature distributions (scale in °C) during RF heating considering an in vivo setting, i.e. including the blood perfusion term, and with a initial temperature of 37°C.

An additional computer simulation was conducted in order to mimic an in vivo scenario, i.e. including the blood perfusion term in Eq. ([1]) and initial and body temperature of 37°C. In spite of including this term, which actually removes heat from the tissue, the computed thermal lesions were larger than in the case with blood perfusion term: 12.2 mm wide and 3.8 mm deep. Figure 9 shows the evolution of the temperature distributions during RF heating in this case. We also simulated an in vivo scenario in which a clamping maneuver is conducted during RF heating, i.e. initial and tissue temperature of 37°C but without the blood perfusion term. The computed thermal lesion was 12.4 wide and 3.8 mm deep.

## Discussion

In this study we developed a mathematical model to describe the electrical and thermal phenomena involved in the use of a TissueLink device. The computer and experimental results were in agreement in terms of thermal lesion depth (2.4 mm in the computer simulations and 2.4 ± 0.6 mm in the experiments). In contrast, we found a disagreement between the theoretical and experimental thermal lesion widths (9.6 mm in the computer simulations vs. 7.8 ± 1.8 mm in the experiments). It is important to point out that the width was measured on the tissue surface, and that in most cases the lesions did not show a completely circular surface geometry, but rather an irregular shape, as shown in Figure 5B (this issue was not considered in the model, and is hence a limitation). Our computer results suggest that the thermal lesion on the tissue surface is mainly created by the effect of the hot saline (Figure 6). We think that the saline flow on the tissue surface is difficult to predict and is also highly dependent on surface irregularities. This would explain the high dispersion in the experimental lesion width values (±1.8 mm) as compared to lesion depth (±0.6 mm) and could suggest an explanation for the discrepancies between the computer and experimental results in terms of thermal lesion width.

This could also be the reason why the impedance progress in the computer simulations matched well with the experiments only in the case of a value of temperature dependence of electrical conductivity (tissue and saline) = +1%/°C. This indicates that the model mimics quite well the physical situation in terms of thermal lesion depth.

Comparing the computer results with and without saline velocity, we can conclude that adding the heat convection term associated with the saline velocity to the governing equation is crucial to keeping the saline layer below 100°C, and hence to modeling the real situation.

As regards the result of the computer simulations mimicking an in vivo scenario, it is interesting to mention that the thermal lesion obtained was larger than ex vivo in both depth and width. This was initially surprising since it is known that including the blood perfusion term in the thermal equation implies a loss of heat so that a smaller thermal lesions is expected when blood perfusion is included. The reason for this result is in the initial temperature, which is significantly higher in the in vivo scenario (37°C vs. 21°C). Since we are modeling a constant voltage protocol, a higher initial temperature implies a lower electrical impedance (265 Ω instead of 310 Ω), and hence a higher amount of delivered power. This reasoning is supported by the fact that an additional computer simulation in which the blood perfusion term was annulled and the initial temperature remained at 37°C produced the same results as in the case of including the blood perfusion term, thus confirming the enormous impact of the initial temperature.

Our computer and experimental findings provide an explanation for the relationship between impedance, electrode insertion depth, and saline layer. In particular, the absence of a saline layer implies the highest impedance value (over 500 Ω). When the saline layer develops (to a height of ≈ 1.5 mm on the electrode surface) impedance drops to around 300 Ω due to the high electrical conductivity of the saline and the greater effective area through which electrical current flows.

Our computer and experimental findings also provide an explanation for the process of creating thermal lesions in the tissue with this type of electrode. Specifically, impedance gradually decreases as heating proceeds, with a drop of around 60 Ω for 20 s. No boiling is observed in the saline for the parameters set (3 mL/min). In the proximity of the electrode, around 1 mm, the thermal lesion is mainly created by the RF power in this same zone, while at a further distance, the lesion is created by the hot saline on the tissue surface by simple thermal conduction and is probably enhanced by the lower saline velocity with distance from the electrode (decays with 1/*r*).

The TissueLink device (Salient Surgical Technologies, Portsmouth, NH, USA) is a dissecting sealer widely used in surgical practice and to date no mathematical models have been proposed to describe this RF electrode. The only model developed for an RF electrode irrigated by saline was proposed by Gopalakrishnan for the case of epicardial RF ablation [21], which also included the heat convection due to saline flow, and the velocity field of the saline was derived from the theory of thin films. Gopalakrishnan focused closely on the mathematical formulation of the saline velocity field in the saline layer and studied the effect of different flow rates on its shape [21]. On the contrary, we were interested in modeling the saline layer as observed in the experiments, i.e. for a specific electrode geometry and with the specific flow rate of one droplet per second, as used in surgical practice. We overcame some limitations of Gopalakrishnan’s study related to the realism of the model. In Gopalakrishnan’s study, all the simulations were stopped when tissue temperature reached 100°C as vaporization was not included in the model. We included not only the vaporization phenomenon but also the temperature dependence of the electrical conductivity, since it is known that its value changes with temperature and it has a strong influence on the results [13]. Finally, we also included the rehydration of the saline previously dehydrated by temperatures around 100°C, to simulate the continuous supply of liquid which impedes total desiccation. It can therefore be said that our model includes some important improvements over Gopalakrishnan’s model.

### Limitations of the study

In our model the tissue was considered to be unaffected both thermally and electrically by the saline irrigation. To solve this limitation, a more complex model could be built, for instance by estimating the amount of saline interstitially infused into the tissue using Darcy’s Law in porous mediums [22], or by considering a specific modification in the electrical conductivity of the tissue caused by the infused saline [23], as has been done in modeling RF ablation with needle-like irrigated (wet) electrodes completely encircled by tissue. However, we considered that saline irrigation of the tissue surface with a saline-linked electrode is a slightly different phenomenon to the saline infusion used in RF ablation with needle-like electrodes. In this case, the saline is directly infused into the tissue (only a small amount escapes through the space between tissue and electrode shaft), and in fact, this infusion is intended to substantially modify the thermal and electrical characteristics of the tissue and consequently to improve RF power deposition [24]. In contrast, in saline-linked electrodes for RF-assisted resection, the saline infusion is basically circumscribed to the tissue surface, and in fact this infusion is intended to keep the tissue surface temperature below 100°C [4] and also to guarantee constant contact between electrode and tissue for efficient delivery of RF power. In this case, unlike the needle-like irrigated electrodes, the saline can move freely on the tissue surface or move away from the electrode, consequently without basically altering the characteristics of the internal tissue.

As regards the RF signal waveform used in experiments and computer simulations, it is important to indicate that, due to the lack of an specific RF generator, the Tissuelink electrode has been historically connected to a conventional electrosurgical unit (ESU), which uses high-voltage signals (kV) and low current with a waveform based on bursts of damped sinusoidal signals (in coagulation mode), and can cause excess of smoke and arcing. In fact, the optimum RF generator for this kind of electrode is not an ESU but an RF generator, as used in RF ablation, i.e. providing a continuous sinus signal with low voltage (100–200 V) and high current (up to 2 A). In other words, the use of an RF generator, as in our study, is the optimum condition for this kind of electrode in terms of absence of smoke and arcing and maximization of coagulation zones. The current tendency is moving towards using a specific RF generator, as employed in our study. In fact, the bipolar version of the Tissue-link electrode (Aquamantys system) simply employs a specific non-modulated sinus signal with low voltage and high current, as we did. In conclusion, the use of a non-modulated sinusoidal signal has and will have further clinical implications. In fact, although our modeling study focused specifically on the Tissuelink Model DS3.0, in essence the mathematical model could be equally valid to study other similar saline-linked RF electrodes, such as the DS3.5-C with a conical point, the floating ball model, or even the bipolar Aquamantys device (Medtronic, Minneapolis, MN, USA).

Since the proposed model had two dimensions, certain issues could not be explained with the computer results, including the lack of symmetry of the top view of the thermal lesion around the electrode (Figure 5B). This could have been due to the irregularities on the tissue surface (dimpled zone) caused during sample preparation, and would imply a preferential direction for the saline flow. In this respect, a three-dimensional model could be developed to study the effect of a non symmetrical supply of saline around the electrode caused by gravity when the electrode is not placed exactly on a horizontal plane (e.g. during a laparoscopic approach), which would mean the saline flows mainly to one side.

Finally, the approach proposed here to model the thermal effect of the continuous supply of saline (equation (5)) is not derived straightforwardly from physical laws, but from assuming that saline vaporization will be permanently compensated and hence the gas phase will never be reached in the saline. A more formal approach could be developed in the future by including a mathematical framework which accurately describes the mutual interaction between saline vaporization and continuous supply in terms of physical phenomena. This more complex approach would make it possible to study the effect of sporadic cessation or drastic reduction in saline flow.

In spite of these limitations, we think that the proposed model could be used in future studies oriented towards suggesting technical improvements of the design and enhancing saline-linked RF electrodes in terms of safety and performance.

## Conclusions

The proposed model mimicked thermal performance during heating in terms of thermal lesion depth. A discrepancy was found about in the lesion width. The model in general provided an explanation for 1) the relationship between impedance, electrode insertion depth and saline layer and 2) the process of creating the thermal lesion in the tissue with this type of electrode, especially the role of the saline layer.
